# Associations of farm management practices with annual milk sales on smallholder dairy farms in Kenya

**DOI:** 10.14202/vetworld.2015.88-96

**Published:** 2015-01-25

**Authors:** Shauna Richards, John VanLeeuwen, Getrude Shepelo, George Karuoya Gitau, Collins Kamunde, Fabienne Uehlinger, Jeff Wichtel

**Affiliations:** 1 Department of Health Management, Atlantic Veterinary College, University of Prince Edward Island, 550 University Avenue, Charlottetown PEI Canada, C1A 4P3; 2 Department of Clinical Studies, Faculty of Veterinary Medicine, University of Nairobi, Nairobi, Kenya; 3 Department of Biomedical Sciences, Atlantic Veterinary College, University of Prince Edward Island, 550 University Avenue, Charlottetown PEI Canada, C1A 4P3; 4 Department of Large Animal Clinical Sciences, Western College of Veterinary Medicine, University of Saskatchewan, 52 Campus Drive, Saskatoon SK Canada, S7N 5B4

**Keywords:** dairy cattle nutrition, management factors, livelihood, smallholder farm

## Abstract

**Aim:**

Cows on smallholder dairy farms (SDF) in developing countries such as Kenya typically produce volumes of milk that are well below their genetic potential. An epidemiological study was conducted to determine reasons for this low milk production, including limited use of best management practices, such as suboptimal nutritional management.

**Methods:**

An observational cross-sectional study of 111 SDF was performed in Nyeri County, Kenya in June of 2013 determining the effect of cow factors, farmer demographics and farm management practices on the volume of milk sold per cow per year (kg milk sold/cow). In particular, the effect of feeding high protein fodder trees and other nutritional management practices were examined.

**Results:**

Approximatly 38% of farmers fed fodder trees, but such feeding was not associated with volume of milk sold per cow, likely due to the low number of fodder trees per farm. Volume of milk sold per cow was positively associated with feeding dairy meal during the month prior to calving, feeding purchased hay during the past year, deworming cows every 4 or more months (as opposed to more regularly), and having dairy farming as the main source of family income. Volume of milk sold per cow was negatively associated with a household size of >5 people and feeding Napier grass at >2 meters in height during the dry season. An interaction between gender of the principal farmer and feed shortages was noted; volume of milk sold per cow was lower when female farmers experienced feed shortages whereas milk sold per cow was unaffected when male farmers experienced feed shortages.

**Conclusions:**

These demographic and management risk factors should be considered by smallholder dairy farmers and their advisors when developing strategies to improve income from milk sales and animal-source food availability for the farming families.

## Introduction

Poverty in developing countries has contributed to chronic undernourishment of 870 million people, or 12.5% of the world population in 2010-2012 [1]. Livestock agriculture is a source of high quality food and income and has a role to play in alleviating poverty and improving human nutrition and health [2,3]. Dairy cattle can provide milk for the families who own them, as well as a source of income through milk sales. Smallholder dairy farms (SDF) of 1-10 cows make up the majority of dairy farms in developing countries such as Kenya [4]. However, many of the owners of SDF in sub-Saharan Africa are limited in their knowledge about a variety of animal husbandry topics, including nutrition [4-6]. Improving the nutrition of dairy cows, specifically lactating cows, could lead to substantial improvement of nutrition in people, while also reducing the effects of poverty through increased income from sold milk.

The Mukurweini Wakulima Dairy Ltd. (MWDL), located in Nyeri County, Kenya, is a dairy group made up of over 6000 SDF members. This area is in an agro-ecologic zone that is well-suited to dairy farming [7]. Membership with MWDL has proven to be beneficial in that it was associated with improved quantity and quality of diets for women and children. Specifically, compared to non-member farmers in the region, MWDL members were found to have higher percentage consumption from animal source foods and greater dietary diversity, as well as lower prevalence of inadequate intake of milk-sourced micronutrients.

On average, MWDL members earn more than $62.50 per month from dairy farming, leading to a yearly dairy farming income of over $750 [8]. Nearby, in Kiambu District, Kenya, the SDF inflation-adjusted net farm income was $294 per year [9]. Low milk production was noted and the authors suggested that this was due to limited feed availability and sub-optimal reproduction.

Yearly milk yield per cow from SDF in Kenya varies from 850-3150 kg/cow [10,11], which is low in comparison to milking cows in more intensive North American dairy farms that produce, on average, 7800 kg/cow [12]. The average daily milk production on SDF in the MWDL, unadjusted for stage of lactation, was 9.2 kg/day or 2806 kg/year for a 305 day lactation [4]. Low production has been attributed to cross-breeding of cattle, poor management of heifers, and poor nutrition of cows [4,10].

Members of MWDL have been reported to feed Napier grass, other grasses, and high protein forages, which are good sources of nutrition; however, they also feed banana leaves which are quite poor in nutritive quality [4,7]. In Limuru District, Kenya, 55% of SDF had inadequate quantities of forage to allow for optimal milk production, and 75% had inadequate quality of forages [13]. Similarly, the quality of purchased commercial feeds was found to be inadequate in providing balanced supplemental nutrition over basal forage diets on 85% of farms, but the quantity of feed fed per cow was not evaluated [13]. Typical early-lactation Holstein cows weighing 454 kg and producing 15 kg of milk per day require 9-10 kg of dry matter intake (DMI) per day, while the same cow in mid-lactation producing 20 kg of milk/day requires 16-17 kg of DMI [14]. To support these levels of milk production, when feeding typical grass or legume forages, nutrient-dense concentrates (e.g. dairy meal) should form 20-60% of the daily ration on a dry matter basis [14]. Thus, typical Holstein cows require 1.8-10.2 kg of dry matter in concentrates per day to support optimal production, depending upon total DMI and stage of lactation. Kenyan SDF typically feed 2 kg of concentrates/cow/day, regardless of the stage of lactation, which suggests inadequate nutrient intake for optimal milk production, particularly during peak lactation [15].

The fodder tree *Calliandra sp*. can be a locally appropriate and beneficial forage source when fed to milking cows in East Africa [16]. It has a protein concentration (30% crude protein on a dry matter basis) similar to that of dairy concentrate feeds. Feeding *Callianda* has improved milk production and thus milk-derived income by $62-122 per year when fed as a substitute or in addition to concentrates [16]. However, this plant is underutilized by farmers due to lack of knowledge about the possible benefits and cultivation requirements, and limited market access to seedlings.

The objective of this study was to evaluate the effects of cow and herd level factors, in particular the effect of feeding high protein fodder trees and other nutritional management factors, on the volume of milk sold in smallholder herds in the Nyeri region.

## Methods

### Ethical approval

This study was approved by the Research Ethics Board and the Animal Care Committee of the University of Prince Edward Island, MWDL, and Farmers Helping Farmers, a partner non-governmental organization. Signed consent of all participants was obtained after the study was fully explained.

### Study site and design

This cross-sectional study of 111 herds was performed in June and July 2013 in the Mukurweini area, Nyeri County, Kenya. Enrolled herds were members of MWDL. Mukurweini has an estimated population of 83,932 people as of 2009, and covers 179 km^2^ [17]. Nyeri County is part of Kenya’s Eastern Highlands spanning an area of 3266 km^2^ [18]. It is located between longitude 36° and 38° east, and between the equator and latitude 1° south [18]. Mount Kenya is located to the east of Nyeri County at an altitude of 5199 m, and the Aberdare Range is to the west at 3999 m [18]. The study area is considered part of the wet medium altitude regions of the humid highlands within an altitude range of 1500 and 2500 m, and where there is annual rainfall of over 1000 mm and humidity >50% [19]. This area is considered to be in agro-climatic zone I that has a high potential for growing crops [19].

### Sampling

A required sample size of 108 farms was calculated, based on unpublished milk production data obtained from a pilot project in 2012 in the same region. One hundred and eleven farms were enrolled to allow for any herd withdrawals from the study. Farms were selected from a database held by MWDL of cows that were artificially inseminated (AI). Of the 6000 eligible MWDL members using AI, herds were considered candidates if they had at least one cow inseminated approximately 9 months prior to the start date of the study, and this cow was confirmed pregnant or had calved <1 week before the scheduled visit date. These criteria were employed because the present observational study was partnered with a prospective study requiring at least one fresh cow per enrolled herd. Farms were excluded if they had more than 5 adult cows, as this is not typical of SDF in this area [4], however, no farms exceeded this size limit. Every eligible farm on this list that was recruited agreed to participate in the study. Farmers who participated in the study received free veterinary care for their cattle on the visit date (as required), as well as deworming of the cow which had most recently calved.

### Data collection

The outcome of interest was volume of milk sold per cow during the last year (kg milk sold/cow), estimated from records of volumes of milk sold per cow during the 12 months prior to the farm visit (i.e. from June 2012-May 2013). These data were collected and made available by MWDL via computerized milk sales records for individual farmers.

A questionnaire comprising open and closed-ended questions was completed in one farm visit for each herd. The data collected contained the following items: household, farm, and cows; dairy income; forage management; and feeding practices. All monetary amounts reported are in US dollars. The gender of the primary farmer was established, as well as the marital status, age and education level of the farmers and spouse, as applicable. Farm size was based on land owned and rented. The number of lactating and dry cattle was determined over the previous 12 months. Farmers were also asked to identify if any cows died in the previous year, and the cause(s) of death, if known.

Farmers were asked closed-ended questions concerning the ration (forages, concentrates, and vitamins/minerals) fed to cows during the last year, with a positive reply indicating that a particular ration item was fed at least once during the past year to one or more cows. Farmers were also asked to specify any feeds offered that were not included in the survey. The farmers were asked to report any shortages of specific feedstuffs during the year, as well as what they stored for feeding during the dry season. Deworming practices were also ascertained.

Farmers’ perceptions were ascertained on how much concentrate (in kg) their typical feed measuring container held. The quantity of concentrate farmers would typically feed a cow on the day that it calved (based on their perception of what their typical measuring container held) was also determined through questioning. The estimates farmers gave were compared to actual feed weights using a weight scale. Farmers were asked if they had changed the amount of concentrates fed to cows during the month prior to calving and during the first 5 months after calving, and if they did, what factors they considered when making these decisions.

Questions pertaining to forage management included: the height at which Napier grass was typically cut for feeding to milking cows during the rainy and dry season; if they fed fodder trees in the last year; if they perceived a net benefit to growing and feeding fodder trees; how many fodder trees they had and of what kind; where fodder trees were planted; and problems they encountered pertaining to the cultivation of fodder trees.

### Survey administration

The face-to-face interviews were conducted on each farm by one of two female veterinarians (SR or GS), with a female translator as required. Survey questions were posed to the identified principal farmer, when available. In most situations, the spouse and farm employees (if any) were present and encouraged to contribute to answering the questions. If discrepancies arose between individuals on the same farm a consensus between them formed the final response.

### Data management and analysis

#### Descriptive statistics

Data were manually entered into Microsoft Excel for Mac 2011 (Microsoft Corporation, 2010). The data were then imported to Stata 12.1 for Mac (StataCorp, 2012), checked for accuracy, and analyzed using descriptive statistics. Proportions positive were determined for categorical variables, and ranges, means, quartiles, and medians were determined for continuous variables.

### Linear regression analysis on factors associated with milk sales

Univariate linear regression of variables was performed to determine unconditional associations with the outcome of interest, which was the square root-transformed milk sold/cow during the last 12 months. This transformation was done to achieve normal distribution of the outcome, and meet model assumptions. Univariate associations with p<0.25 were eligible for the following multivariable linear regression analyses.

Multivariable linear regression was performed to determine factors associated with the square root-transformed volume of milk sold (per cow/year), while controlling for possible confounding among model variables. Variables were removed if significance was >0.05 p-value, unless confounding was present. Interaction terms for all variables in the final model were evaluated for their significance, as well as possible confounding. The final model was evaluated by looking at standardized residuals, leverage, difference in fits and delta-betas to ensure model assumptions were met.

## Results

### Descriptive results

Tables 1-5 show descriptive results for the various cow and farm level variables for the participating farms. Gender of the primary farmer was equally distributed, with women tending to have a lower level of education when compared to men (Table 1). Nearly one-third of farms had 5 or more household members (Table 1). The median age of female and male farmers were 48 and 51 years, respectively (Table 2). The median volume of milk sold was 768.7 kg/cow/year.

**Table-1:** Farm level descriptive statistics of demographic categorical variables for 111 smallholder Kenyan dairy farms in 2013

Variables	Category	Number	Proportion (%)
Gender	Female	56	50.5
	Male	55	49.5
Marital status	Married	94	84.7
	Widowed	15	13.5
	Single	2	1.8
Women’s education level	None	11	10.3
	Primary	57	53.2
	Secondary	37	34.6
	College/University	2	1.9
Men’s education level	None	6	6.1
	Primary	45	45.5
	Secondary	43	43.4
	College/University	5	5.0
Membership duration at MWDL	1-3 years	13	11.7
	4-6 years	12	10.8
	7-9 years	15	13.5
	10+years	71	64.0
Percent of income from dairy farming	<50%	42	38.5
	50-70%	38	34.9
	>70%	29	26.6
Number of people living in household	1	5	4.5
	2	22	19.8
	3	22	19.8
	4	29	26.2
	5	20	18.0
	6+	13	11.7

MWDL=Mukurwe-ini Wakulima Dairy Ltd.

**Table-2:** Farm level descriptive statistics for demographic and management continuous variables for 111 smallholder Kenyan dairy farms in 2013

Variable	Median	Range	Number
Women’s age (years)	48.0	19-83	107
Men’s age (husband or son - years)	51.0	22-84	99
Area of land owned (acres)	1.9^a^	0.1-9	111
Area of land rented (acres)	0.0	0-8.3	111
Actual weight of perceived standard 2 kg measuring container for grain (kg)	1.5	0.5-3.5	111
Dairy meal fed to cow on day of calving (kg)	2.0	0.0-8.0	111
Other grain fed to cow on day of calving (kg)	0.0	0.0-5.0	111
Average number of cows/herd	1.5	1-4.5	111
Yearly Milk Sold per Cow (kg)^b^	768.7	14.5-3013.9	99

a Mean reported for normally distributed data,

b Only 99 of 111 farms had consistent milk production records on file for the last 12 months

**Table-3:** Cow level descriptive statistics of lactating cow feeding practices over the last year for 110 smallholder Kenyan dairy farms in 2013

Variable	Number of farmers feeding	Proportion (%)
Napier grass	110	100.0
Grass silage	6	5.5
Maize silage	3	2.7
Purchased hay	41	37.2
Home grown hay	56	50.9
*Desmodium*	43	39.1
Sweet potato vines	82	74.6
Other high protein fodder	13	11.8
Tree fodders	42	38.2
Banana leaves	101	91.8
Other fodder	72	65.5
Dairy meal	96	87.3
Wheat bran	67	60.9
Maize germ	81	73.6
Other grain	52	47.3
Vitamin/mineral powder	107	97.3
Vitamin/mineral block	86	78.1

**Table-4:** Descriptive statistics of categorical feeding practices variables for 111 smallholder Kenyan dairy farms in 2013

Variable	Number of farmers		Proportion (%)	
Seasonal height of Napier grass	Rainy	Dry	Rainy	Dry
<1 m	21	54	18.9	48.7
>1 m<1.5 m	54	32	48.7	28.8
>1.5 m<2 m	15	13	13.5	11.7
>2 m	21	12	18.9	10.8
Shortage of feed (s)^a^				
Forage	67		60.4	
Grains	28		25.2	
Vitamins/minerals	13		11.7	
Water	1		0.9	
Storage of feed (s)^a^				
Grass hay	8		7.2	
Silage	2		1.8	
Maize stover	11		9.9	
Other	4		3.6	
Dairy meal fed in month prior to calving				
Yes	75		67.6	
No	36		32.4	
Change of amount of dairy meal fed prior to calving				
Yes	39		52.0	
No	35		48.0	
Increase or decrease amount of dairy meal fed prior to calving				
Increase	34		87.2	
Decrease	5		12.8	
Vitamin/mineral fed prior to calving	106		95.5	
Dry cow mix	17		16.0	
Lactating cow mix	56		52.8	
Block	11		10.4	
Unsure	22		20.8	
Frequency of deworming				
>Every 3 months	81		73.0	
Less often	30		27.0	
Change of amount of dairy meal fed in the first 2 months post calving^a^				
Yes	46		41.4	
No	65		58.6	
Two most important factors considered when changing amount of dairy meal fed in first 2 months post-calving^a^				
Cows yield	40		43.5	
Affordability	15		16.3	
Availability	8		8.7	
Month of lactation	8		8.7	
Other	7		22.6	

a These variables allowed farmers to choose more than one answer if more than one answer was applicable to them

**Table-5:** Descriptive statistics regarding the growth, use, and perception of fodder trees by 110 smallholder Kenyan dairy farmers in 2013

Categorical variables	Number	Proportion (%)
Perceived benefit of fodder trees		
Yes	54	49.1
No	25	22.7
Don’t know	31	28.2
Farms which grow fodder trees		
Yes	38	34.6
No	72	65.4
Source of trees^a^		
Gift	16	42.1
Purchased	12	31.6
Other	10	26.3
Location where trees are planted^a^		
Randomly	13	34.2
Boundaries	12	31.6
Slopes	10	26.3
Inter-planted	4	10.5
Other	2	5.3
How tree leaves are fed^a^		
All cows/calves	25	65.8
By milk production	12	31.6
By age	1	2.6
Perceived benefits of trees^a^		
More milk produced	32	84.2
Healthier cow	18	47.4
Stakes and fuel source	10	26.3
Lower feed costs	7	18.4
Other	5	13.1
Perceived problems with trees^a^		
Yes	10	26.3
No	28	73.7
What problems were perceived^a^		
Dries up	3	7.9
Eaten by other animals	3	7.9
Difficult to grow as seedlings	2	5.3
Other	2	5.3

**Continuous variables**	**Measured statistic (n=38)**	

Number of *Calliandra* trees per farm that had trees		
Mean	117	
Median	6	
Range	1-1500	
Year when trees were first planted		
Median	2008	
Range	1994-2013	

a These variables allowed farmers to choose more than one answer if more than one answer was applicable to them

One of the 111 farmers had only one recently calved heifer, therefore this farmer could not report feeding practices over the previous year for a cow. Over 50% of farmers fed Napier grass, sweet potato vines, home-grown hay, banana leaves, other fodder such as weeds and waste from crops, dairy meal, wheat bran, maize germ, and vitamin and mineral powder or block, while purchased hay was fed on one-third of farms (Table 3).

Farmers tended to feed Napier grass cut at shorter heights during the dry season in comparison to the rainy season (Table 4). 60% of farmers reported a shortage of forages in the past year, with less than one-quarter of farmers stored any feed. Two-thirds of farmers fed dairy meal to their cows in the month prior to calving, but less than half of those farmers increased amounts of dairy meal fed to cows approaching the date of calving (Table 4).

Almost half of farmers perceived a benefit of cultivating fodder trees such as *Calliandra*, but only one third of SDF owners grew *Calliandra* fodder trees in the last year (Table 5). Three farmers planted an alternate fodder tree, Mulberry; however, they grew *Calliandra* as well.

### Associations with volume of milk sold

Of the 111 farmers enrolled, 99 were found to have complete historical milk sales records for the period of interest, and 98 of these had complete questionnaire data; therefore, the results of analytical statistics involving milk sales are based on 98 (multivariable regression) or 99 (univariate regression) farms.

In the 99 herds with complete milk sales, 15 variables were found to be unconditionally associated with volume of milk sold at p<0.25 (Table 6). Eight of these variables were retained in the final multivariable linear regression model (Table 7). While accounting for confounding in the multivariable model, volume of milk sold per cow was positively associated with feeding dairy meal during the month prior to calving, feeding purchased hay during the past year, deworming cows every 4 or more months (as opposed to more regularly), and having dairy farming as the main source of family income. Volume of milk sold per cow was negatively associated with a household size of >5 people and feeding Napier grass at >2 m in height during the dry season. While 38% of farmers fed fodder trees, such feeding was not associated with volume of milk sold per cow. An interaction between gender of the principal farmer and feed availability was found, such that volume of milk sold was lower when female farmers experienced feed shortages, whereas milk sold per cow was unaffected when male farmers experienced feed shortages (p=0.029). With other variables held constant, for a female farmer experiencing a feed shortage, predicted yearly milk sold/cow was 62.4 kg/cow/year lower when compared with the same farmer experiencing no feed shortage; for a male farmer experiencing a feed shortage, predicted yearly milk sold/cow was 20.2 kg/cow/year higher when compared to the same farmer experiencing no feed shortage. The 8 variables and interaction in the final model explained 28.3% of the variation in yearly milk sold/cow. (Adjusted R-squared was 20.9%).

**Table-6:** Univariate linear regression results of variables marginally associated (p<0.25), or of a priori interest with respect to possible confounding, with yearly milk sold/cow for 99 smallholder Kenyan dairy farms in 2013

Variables	Variable range (unit)	Change in milk sold/cow/year (kg)^a^	*P* value
Average cows/herd^b^	1,4.5 (cows)	7.4	0.228
Household size	<5, ≥5 (people)	−21.2	0.043
Gender	0,1 (female, male)	4.2	0.327
Income from dairy	<50, ≥50 (%)	23.1	0.057
Fed purchased hay	0,1 (no, yes)	19.2	0.042
Fed home-grown hay	0,1 (no, yes)	16.2	0.054
Fed *Desmodium*	0,1 (no, yes)	10.7	0.130
Fed high protein fodder	0,1 (no, yes)	22.1	0.159
Napier grass height fed in rainy season	<2, ≥2 (meters)	−10.9	0.141
Fed maize germ	0,1 (no, yes)	18.3	0.072
Fed other grains	0,1 (no, yes)	20.8	0.029
Fed dairy meal in month prior to calving	0,1 (no, yes)	9.1	0.189
Feed shortage in last year	0,1 (no, yes)	10.5	0.131
Fed vitamin/mineral powder	0,1 (no, yes)	108.2	0.163
Infrequent cow deworming	0,1 (> every 3 month, less often than every 3 month)	13.0	0.134

Outcome of milk sold/cow/year is square root transformed,

a Change in milk sold/cow/year for categorical variables refers to the change when the variable is present (2^nd^ value listed for ‘Variable range’), whereas for continuous variables, it refers to the change going from the 25^th^ percentile to the 75^th^ percentile,

b Continuous variable of the average number of adult cows in a herd during the year, adjusting for any deaths in the herd

**Table-7:** Multivariable linear regression results of variables associated with yearly milk sold/cow for 98 smallholder Kenyan dairy farms in 2013

Variables	Coefficient^a^ (95% CI)	P-value	Change in milk sold/cow/year (kg)
Household≥5 people	−4.10 (−8.18, −0.02)	0.049	−16.8
Income≥50% from dairy	4.79 (0.94, 8.64)	0.015	22.9
Gender of farmer is male	−4.55 (−10.93, 1.82)	0.159	−20.7
Feed shortage in last year	−7.90 (-13.92, −1.87)	0.011	−62.4
Male farmer with feed shortage (interaction)	9.09 (0.95, 17.22)	0.029	82.6
Napier grass height (>2 meters) fed to cows in rainy season	−4.61 (−8.88, −0.33)	0.035	−21.3
Purchase hay for cows	4.05 (0.01, 8.09)	0.049	16.4
Dairy meal fed to cows in month prior to calving	5.00 (0.53, 9.47)	0.029	25.0
Deworm cows less frequently then every 3 months	6.01 (1.48, 10.54)	0.010	36.1
Constant	21.56 (11.59, 31.58)	<0.001	

a Coefficients on square root transformed scale, CI: Confidence interval

## Discussion

The final model from this observational study identified and quantified the 8 most significant demographic and management factors that were associated with the volume of milk sold per cow on the 98 farms with complete milk sales and management data. Four of these 8 factors were related to nutrition (shortage, napier height, close up feeding, and hay). This is the first study to identify that the association between feed shortage and milk sold per cow depended on the gender of the primary farmer, with herds managed primarily by women having substantially lower volume of milk sold when experiencing a feed shortage. A feed shortage had no negative effect when the primary farmer was male (Table 7). This gender difference might be explained by the fact that men typically have more control over the household income in Kenya when compared with women, and therefore, men may be more able than women to purchase additional feeds during times of feed shortages [20]. Men may also have more free time available to search for forages in times of feed shortages, whereas women may not. In addition, this interaction could be due to women retaining milk for family nutrition rather than for sale, and directing income to supporting the family as opposed to feeding cattle in times of feed shortages. Feed shortages were common, with 60% of farmers reporting a shortage in forage (70% and 51% of female and male farmers reported shortages, respectively), and 92% of farmers resorting to feeding banana leaves which are a very poor forage for a milking cow [7].

Several other nutritional management factors were also significantly associated with the outcome variable (Table 7). The volume of milk sold annually per cow was lower when feeding Napier grass over 2 m in height in the rainy season. Tall Napier grass is high in fiber and low in protein and energy compared with shorter Napier grass [7]. Conversely, feeding purchased hay had a positive effect on milk volume sold per cow. This could be due to cows producing more milk when provided with high quality forage in times of drought, and/or because farmers with sufficient resources to buy additional feeds were able to manage a farm at a higher milk production level. Additionally, farmers who fed dairy meal to their cows in the month prior to calving (68% of farmers - Table 4) had increased volumes of milk sold. This positive association is not unexpected when considering that under circumstances of sub-optimal nutrition, improving nutrition generally leads to higher milk production in dairy cattle [7], and more specifically, feeding nutrient-dense concentrates during the close-up period allows the rumen flora to adjust to a higher plane of nutrition prior to calving so the cow’s body (digestive, renal, mammary systems) is able to produce higher volumes of milk post-calving [14].

In addition, nearly half of the dry cows receiving dairy meal were also receiving increased amounts of dairy meal as they got closer to calving, indicating that a third of farmers likely understood the concept of “steaming up” to improve early lactation yields [14]. However, while most farmers (97%) were feeding vitamin and mineral supplements to their lactating cows (Table 3), they often (53%) fed a lactating cow mineral to dry cows (Table 4), which could increase the risk of metabolic conditions such as milk fever in higher producing cows.

Farmers fed a median of 2 kg of dairy meal concentrate on the day of calving and very little of other types of concentrates (Table 2), which is similar to the findings which described farmers feeding 2 kg of concentrate per day regardless of stage of lactation [15]. Only 41% of farmers changed the amount of dairy meal they fed their cows in the first 2 months post-calving (Table 4), which suggests that most cows are not being fed based on production. The farmers who did alter their feeding in the first two months post-calving reported that the two most important considerations to change the amount of concentrate being fed were a cow’s milk yield and affordability of feed. This demonstrates an understanding of the lactation curve, but also that cost of feed was an important driving force affecting purchasing habits among smallholder dairy farmers.

The vast majority (87%) of farmers fed cows dairy meal concentrate in the past year (Table 3), while feeding wheat bran and maize germ was also common (61 and 74%, respectively). However, 54% of farmers were found to improperly measure concentrate portions fed, assuming that a standard 2 kg plastic container (which held oil) would hold 1.5 or 2 kg of concentrate, when in fact it held closer to 1.25 kg. This improper measurement resulted in unintended underfeeding of the cows by those MWDL farmers. However, this unintended underfeeding was not a significant factor in the final model.

Overall, 38% of farmers fed fodder trees but such feeding was not associated with volume of milk sold per cow. The reasons for this lack of association could be because only a third of farmers had *Calliandra* fodder shrubs, and those farmers with *Calliandra* had a small number of shrubs and indiscriminately fed it, regardless of the age or stage of lactation of their cattle (Table 5). Only 49% of farmers perceived a benefit to planting fodder trees, with 28% unaware of any benefit of fodder trees in general. Of the farmers that did plant *Calliandra*, it appeared there was limited understanding of optimal planting and feeding practices. Farmers tended to see the cultivation of fodder trees as being in competition for land that would normally be used for other crops, rather than planting *Calliandra* trees in areas that were currently unproductive, such as boundaries which may be lined with trees, or currently had shrubs that could not be fed to cattle. Feeding *Calliandra* to all animals in the limited quantities being grown on-farm would also lead to lower milk production benefits than if they were primarily or exclusively fed to early lactating cows or young heifers. Many farmers voiced concerns with access to purchasing seedlings, with only 32% of the 38 farmers with *Calliandra* trees having purchased them. The farmers that were unaware of the benefits of *Calliandra* also voiced concerns about where to purchase these seedlings after they were provided with education on the benefits of them. Although a minority of farmers had *Calliandra* shrubs, a majority of farmers (75%) were feeding another high protein forage, sweet potato vines (Table 3). However, fewer farmers were feeding *Desmodium* (39%).

Deworming frequency was one of the three non-nutritional management factors in the final model of milk sold per cow. Farmers that reported deworming their cows every 3 months or more often had less volume of milk sold per cow than those deworming less frequently (Table 7). This result may seem counter-intuitive because cows with a lower parasite burden tend to have better milk production [21], and this would be expected to lead to higher milk sales. The negative relationship between frequency of deworming and apparent milk production in this study may be a function of the chosen outcome variable; farmers who deworm their cows more frequently will have larger volumes of milk withdrawn from human consumption and thus lower volumes available for sale because virtually all dewormers for sale in Kenya currently have a milk withdrawal period. Another explanation for this counter-intuitive finding may be that farmers may under-dose with the dewormer because of inaccuracies in estimating the weights of cows, especially the heavier cows such as Holstein cross-breeds (the authors noted this often among farmers). Chronic under-dosing of dewormer has been associated with parasite resistance [22], and could result in resistant populations of worms, leading to lower milk production in the affected cows. Farmers may also deworm sick cows more regularly, even though parasitism may not be the cause of illness.

Farmers with 5 or more people living at home had lower volumes of milk sold per cow in the last year than those with fewer people living at home (Table 7). This could be due to the fact that larger families consume more of the milk at home instead of selling it. Farms that had a majority of their income from dairy farming also sold greater volumes of milk per cow (Table 7). A similar finding was reported where farms that were more dependent on non-farm income tended to have poorer milk production on their dairy farms [23]. Therefore, farmers that focus more on dairy farming as opposed to other income sources appear to have better producing dairy farms.

With respect to education, 64% of female farmers and 52% of male farmers had no or only primary level education (Table 1). Farmers with higher levels of education have been reported to have improved production from their dairy farms, likely because higher education has been associated with provision of higher quality feeds [13]. However, the effects of education on our outcome variable were not apparent in our study. This may be due to the fact that the majority of farmers (64%) were members of MWDL for 10 or more years, and MWDL members were shown to be dissimilar to non-members or early onset members; MWDL members have improved milk production, larger herd sizes, greater percent of total income from dairy farming, and improved food security [8].

Despite having a higher proportion of long-term MWDL members in our study population, the median yearly milk sold was 768.7 kg/cow/year, which is low in comparison to previously reported yearly milk production output of 850-3150 kg/cow in Kenya [11]. The discrepancy may be due to the fact that the volume of milk sold is less than that of actual production, as the family would keep some milk for consumption at home. Even more milk would be kept for home use with large households, as shown in our model results where SDF with >5 people living in the home sold less milk than those with fewer residents. Milk sales records were used in our study instead of production records since few farmers keep production records, making sales records the next best available option. The correlation between sales and production records has not been evaluated, and therefore, extrapolation between the findings of the milk sales multivariable model to milk production should be considered with a high degree of caution.

Limitations of our study include the relatively small number of farms to detect many significant associations with the farm level outcome. Only 99 of the 111 study farms had complete sales records for the past year, and due to missing data from one farm (missing the percentage of income from dairy farming), the regression analysis only included 98 farms. Reasons for lack of complete sales records included: (1) farmers having only 1 cow that was dry or producing so little milk that they consumed all their milk at home instead of selling it; (2) farmers only having a recently calved heifer so they would not have any previous milk production records; or (3) farmers just recently became MWDL members. This observational study was partnered with a prospective study requiring 108 farms and at least one fresh cow per enrolled herd, therefore the 12 farms (representing only 11% of the farms) with incomplete milk sales data were allowed to participate in the overall study, and were included in the descriptive statistics of the present study.

Members of MWDL were used exclusively in this study because the prospective study required a fresh cow, which was determined through the computerized records system used by the AI services from the MWDL veterinary unit. Farmers that used other or no veterinary or AI services were therefore excluded, and this may have biased our sampling towards herds with better management practices. However, our results are likely to be representative of areas where the majority of farmers are members of a dairy group similar to MWDL and have farm sizes of 1-4 cows. The household demographics were similar to those also found among members of MWDL [8]. Farm and herd sizes were small, a finding typical of the densely populated Kenyan highlands.

## Conclusions

Volume of milk sold per cow was positively associated with feeding dairy meal during the month prior to calving, feeding purchased hay during the past year, deworming cows every 4 or more months (as opposed to more regularly), and having dairy farming as the main source of family income. Volume of milk sold per cow was negatively associated with a household size of >5 people and feeding Napier grass at >2 m in height during the dry season. An interaction between gender of the principal farmer and feed shortages was noted; volume of milk sold per cow was lower when female farmers experienced feed shortages whereas milk sold per cow was unaffected when male farmers experienced feed shortages. These factors should be considered by smallholder dairy farmers and their advisors when developing strategies to improve income from milk sales.

## Authors’ Contributions

SR, GS, and JV conducted the field data collection. SR and JV wrote the draft manuscript. All authors were involved in the preparation of data collection materials, and the revision and approval of the final manuscript. SR, JV, GG and JW were involved in funding acquisition. SR, JV, GG, GS, JW, FU, and CK were all involved in formulating the study design and methods of implementation and approving the final manuscript. All authors read and approved the final manuscript.
